# Nano-Sized Cyclodextrin-Based Molecularly Imprinted Polymer Adsorbents for Perfluorinated Compounds—A Mini-Review

**DOI:** 10.3390/nano5020981

**Published:** 2015-06-04

**Authors:** Abdalla H. Karoyo, Lee D. Wilson

**Affiliations:** Department of Chemistry, University of Saskatchewan, Saskatoon, S7N 5C9, Canada; E-Mail: abk726@mail.usask.ca

**Keywords:** cyclodextrin (CD), molecularly imprinted polymers (MIPs), adsorption, perfluorinated compounds

## Abstract

Recent efforts have been directed towards the design of efficient and contaminant selective remediation technology for the removal of perfluorinated compounds (PFCs) from soils, sediments, and aquatic environments. While there is a general consensus on adsorption-based processes as the most suitable methodology for the removal of PFCs from aquatic environments, challenges exist regarding the optimal materials design of sorbents for selective uptake of PFCs. This article reviews the sorptive uptake of PFCs using cyclodextrin (CD)-based polymer adsorbents with nano- to micron-sized structural attributes. The relationship between synthesis of adsorbent materials and their structure relate to the overall sorption properties. Hence, the adsorptive uptake properties of CD-based molecularly imprinted polymers (CD-MIPs) are reviewed and compared with conventional MIPs. Further comparison is made with non-imprinted polymers (NIPs) that are based on cross-linking of pre-polymer units such as chitosan with epichlorohydrin in the absence of a molecular template. In general, MIPs offer the advantage of selectivity, chemical tunability, high stability and mechanical strength, ease of regeneration, and overall lower cost compared to NIPs. In particular, CD-MIPs offer the added advantage of possessing multiple binding sites with unique physicochemical properties such as tunable surface properties and morphology that may vary considerably. This mini-review provides a rationale for the design of unique polymer adsorbent materials that employ an intrinsic porogen via incorporation of a macrocyclic compound in the polymer framework to afford adsorbent materials with tunable physicochemical properties and unique nanostructure properties.

## 1. Introduction

Perfluorinated compounds (PFCs) have emerged among the most researched chemicals following the recent classification by the Stockholm Convention on persistent organic pollutants (POPs) [1]. Perfluorooctane sulfonate (PFOS) and perfluorooctanoic acid (PFOA) are among the most commonly studied PFCs [2,3]. PFCs possess unique physicochemical properties as compared to their hydrocarbon counterparts. They are generally apolar, have variable water solubility, low volatility and are relatively stable under extreme conditions. Perfluorinated chemicals have been detected in drinking water, ground water and surface water supplies [2,4], industrial wastewater effluents [5,6] soil and sediments [7], and other aquatic environments [8,9]. PFCs have been associated with wide ranging health risks, such as reproductive and developmental disorders [10,11,12,13,14,15,16], suppression of immunity [17,18,19] and cancer [20,21,22,23]. Although many countries, including Canada and the USA, have restricted the use of PFOA, PFOS and related PFC chemicals, their use in the electroplating and textile industry continues unabated elsewhere [23,24]. The recalcitrant nature of PFCs and their ubiquitous occurrence in the global environment illustrates the need to develop reliable remediation techniques to control their fate and transport, and to mitigate their associated health risks in aquatic environments.

Most of the research reports on PFCs have focused on their physicochemical and toxicological properties [7,25,26,27], fate and distribution [28,29,30,31], and complexation phenomena [32,33,34,35,36,37,38,39,40]. In a recent report by Du *et al.* [41], the removal of PFCs from aquatic and terrestrial environments was reviewed. In general, PFCs are not amenable to conventional chemical treatment (e.g., hydrolysis, photolysis, oxidation and reduction) or biological treatment (e.g., microbial degradation and metabolism) due to their relative inert nature owing to the high stability of the C-F bonds [26,42]. Alternative approaches for the removal of PFCs was reported in the literature that employed ultrasonic [43], ultraviolet (UV) irradiation [44], and oxidation methods [45]. However, the use of such technologies is limited due to their restrictive infrastructure cost, consumptive energy demands and operational time requirements. Adsorption is a physical process that offers a suitable alternative for the physical removal of perfluorinated contaminants from water. Adsorption of PFCs using activated carbon (AC) is well documented with several recent reports [46,47,48,49,50,51,52,53,54,55]. AC-based materials offer a practical approach to the removal of PFCs due to the facile utility of adsorptive-based processes. The practical utility of adsorption-based remediation of aquatic contaminants is supported by the technical simplicity and its relatively low cost compared to conventional chemical-based removal methods [56]. However, the regeneration of carbonaceous materials using chemical and biological methods, oxidation, ultrasound, heat, and solvent assisted methods may be impractical due to the prohibitive economic costs [57,58,59,60,61]. Recently, Yu *et al.* [62] reviewed carbon nanotubes (CNTs) as potential adsorbent nanomaterials for remediation of organic contaminants including PFCs. In spite of their relatively high surface area (~150–1500 m^2^.g^−1^), CNTs were found to have reduced uptake of PFCs due to the limited availability of active sites and weak interactions attributed to their inert character [54,62]. Mineral-based adsorbents such as activated alumina, silica, and zeolite were found to be inferior to AC materials; whereas, commercial ion-exchange resins display improved adsorption but may be limited due to their high cost and the disadvantages associated with regeneration processes that use toxic organic solvents [41].

**Figure 1:**
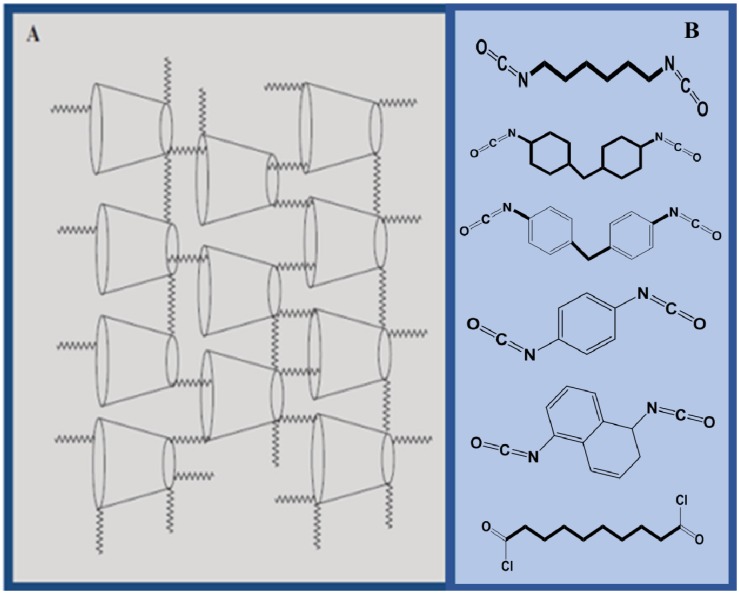
(**A**) A generalized 2-D framework structure of a β-cyclodextrin (β-CD) cross-linked polymer; where the toroids represent the porogen (β-CD) sites and the zigzag line segments are the bifunctional cross-linker scaffold of the framework. The polymer framework is shown as a semi-ordered material where the interior of the toroids are referred to as the “inclusion sites” and the other voids are the interstitial or “non-inclusion sites”; (**B**) Choice of different bifunctional cross-linker units (top-to-bottom; 1,6-hexamethylene diisocyanate (HDI), 4,4'-diicyclohexylmethane diisocyanate (CDI), 4,4'-diphenylmethane diisocyanate (MDI), 1,4-phenylene diisocyanate (PDI), 1,5-naphthalene diisocyanate (NDI), terephthaloyl chloride and sebacoyl chloride) gives rise to materials with variable physicochemical properties (redrawn from [73,74]).

Solid phase extraction (SPE) methods with synthetic polymers reveal uptake properties that compare favorably to high surface area carbonaceous adsorbents, such as AC and CNTs. SPE methods using CD-based polymers have been reported for the efficient removal of organic dyes [63,64,65,66] and other organic pollutants [67,68,69]. Molecularly imprinted polymers (MIPs) offer the advantage of greater selectivity through the appropriate choice of an imprinting template and efficient imprinting conditions. Continued interest in MIPs and non-imprinted polymers (NIPs) may be due to the tunable accessibility of their active adsorption sites, ease of regeneration, and relatively low materials design cost for their synthesis. In this mini-review, an emphasis will be placed on the use of intrinsic molecular imprints that are afforded by the incorporation of macromolecules such as cyclodextrins (CDs) with host cavities that serve as porogen sites. This approach is related conceptually but it is practically different than the preparation of conventional MIPs since the presence of a macrocyclic host in the 3-D framework structure of a cross-linked polymer affords multiple binding sites according to the materials design strategy. Previous reports [70,71,72,73,74,75,76] indicate that the formation of cross-linked polymers containing a macrocyclic host that enables tuning of the surface chemistry and morphology (micron to nanometer scale range) of such porous materials. The two properties, *i.e.*, surface chemistry and morphology, can be tuned systematically by varying factors such as the nature (functionality) of the cross-linker unit, as depicted in Figure 1. A unique feature of macromolecular-based imprinted polymers lies in their impressive host-guest chemistry and related applications in sensor technology, separation science, and diagnostic materials due to their unique molecular recognition properties [77].

Herein, we review the materials design, physicochemical characteristics and performance of different nano-structured MIP adsorbents. Firstly, conventional MIPs are compared with non-imprinted polymer (NIP) adsorbents that are based on pre-polymer scaffolds such as chitosan with a cross-linker, such as epichlorohydrin, to generate modified biomaterial adsorbents for uptake of PFCs. Secondly, variable design strategies and types of CD-based MIPs (CD-MIPs) are highlighted and their physicochemical properties are reviewed and compared with conventional MIP systems. Cyclodextrins (CDs) are macrocyclic (1→4)-linked oligomers of α-D-glucopyranose. Among the most commonly studied CDs are α-, β-, and γ-CD, consisting of 6-, 7-, and 8-glucopyranose units, respectively [78]. Research on MIPs for the sorptive uptake of PFCs is sparse, especially studies that explore the unique structural features of porogen-based macrocycles (such as CDs) for the materials design of MIPs [79]. This review will describe the knowledge gaps that exist for different types of synthetic polymer adsorbents and the design of new materials for the removal of PFCs. An overview of studies that report novel materials which contain macromolecular units for the efficient removal of PFCs will be emphasized.

## 2. CD/PFC Host-Guest Chemistry

Among the various cyclodextrins (CDs), β-CD has the remarkable ability of forming stable inclusion complexes with apolar guests, such as perfluorinated alkanes, in part, due to the good “*size-fit*” complementarity between the guest and the host [80]. In order to form a stable complex, the guest molecules must fit, at least partly, into the CD cavity [81,82]. Apart from the steric considerations, other factors, such as the release of high energy water, hydrophobic effects, and a range of intermolecular interactions (e.g., dispersion, dipole-dipole, electrostatic, and hydrogen bonding) may contribute to the stability of the complex formed between a CD host and a perfluorinated guest [83]. Thus, the stability of CD inclusion compounds in aqueous solution depends on the degree to which the cavity is occupied by the hydrophobic part of the guest and the various stabilizing hydration forces. Complexes between β-CD and PFCs of variable chain length were concluded to form stable inclusion complexes in aqueous solution, according to ^19^F/^1^H NMR spectroscopy [32,33,34,35], viscometry [32,33,34,35,36], conductometry [37,38,39], and sound velocity [40]. Complexes with greater stability were reported for CDs with C8 perfluorocarbon (PFC) guests (10^2^ M^−1^ to 10^4^ M^−1^) [32,33], as compared with their hydrocarbon analogues (≈ 10^2^ M^−1^) [84]. Moreover, Xing *et al.* [33] have shown that β-CD preferentially includes a fluorinated guest when exposed to a mixture of sodium perfluorooctanoate and sodium alkyl sulfate.

Wenz has reviewed the utility of CDs as versatile building blocks for supramolecular structures because they can be linked regioselectively to multiple substituents, both covalently and non-covalently [85]. Studies of polymer materials, especially CD-based copolymers, with simple organic/inorganic model compounds contribute to an understanding of sorption based processes and applications [71]. CD-based materials extend the application of the host-guest chemistry of CDs through their use as building blocks in materials design. CD-based polymers offer unique opportunities to engineer new materials with tunable properties, as follows: (1) surface area, (2) pore structure, (3) solubility, (4) chemical stability (regeneration), and (5) surface functionality of the sorbent, affording optimal sorption properties. The scarcity of research reports related to the preparation and use of synthetic polymer materials for the removal of fluorinated contaminants is attributed to numerous challenges associated with materials design to afford efficient adsorption of such chemicals and possibly overcome the disadvantages of carbonaceous adsorbent materials. Some challenges related to materials design include: (1) incompatibility of fluorocarbon (FC) guests with hydrocarbon (HC) rich host materials, (2) competition for the adsorbent from water and other organic molecules in the aqueous matrix, and (3) challenges associated with controlling the surface area and pore structure properties to afford improved accessibility of the active adsorption sites. The phase separation of FCs with HCs was described by Shatha *et al*. [86] and Asakawa *et al*. [87]. Furthermore, interaction of adsorbents with water or other competing organic molecules can substantially lower the overall affinity of the target molecules with the adsorbent. Efficient adsorbents for PFCs can be afforded by enhancing molecular recognition of the guest in a controlled manner, in addition to controlling the relative surface area and accessibility of the active sites. This can be achieved by controlling the functionality of the monomer units, monomer composition, and type of the cross-linker, respectively, as depicted in Figure 1, and in agreement with studies of solid phase extraction (SPE) materials [88]. In general, improved molecular recognition of target fluorinated substrates can be achieved through the incorporation of macrocyclic CD porogens within the 3-D polymer framework, in accordance with the unique host-guest chemistry of such materials.

## 3. Adsorbents for PFCs

### 3.1. NIP Adsorbents

The challenges associated with the design of polymer adsorbent materials for targeting PFCs was outlined in Section 2. Therefore, a directed materials design strategy should address the challenges associated with fluorocarbon-hydrocarbon (FC-HC) incompatibility whilst ensuring that the materials possess ready accessibility to the adsorbent binding sites. Materials with suitable surface area and textural porosity with pore dimensions that range in size from nano- to micron-size may offer enhanced adsorption properties. More importantly, the surface chemistry of the produced materials should afford good selectivity towards the target molecules in order to discriminate between other organic molecules of similar size and shape.

Studies of NIP-based materials for the adsorption of PFCs were reviewed by Du *et al*. [41] that described biomaterial adsorbents. Zhang *et al*. [89] have reported the preparation of NIP adsorbent materials with chitosan beads cross-linked with epichlorohydrin (ECH) for the uptake of PFOS. The chitosan-based adsorbent was reported to have high sorption capacity of ~5.5 mmol.g^−1^ (pH 3, 100 h), ~1.72 mmol.g^−1^ (pH 7.0, 24 h), and ~0.72 mmol.g^−1^ (pH 9.5, 10 h). The higher uptake of the anionic form of PFOS by the chitosan adsorbent surface was driven by ion exchange interactions at acidic pH conditions due to the protonation of the amine groups of chitosan. The slow adsorption kinetics was evidenced by the longer equilibration times that may restrict usage of such materials as practical sorbent materials. Recently, Deng and coworkers grafted long polymers with amine groups onto nonporous biomaterials, such as cotton [90] and rice husk [91], to target PFOA, PFOS, and perfluorobutyric acid (PFBA). By analogy to the chitosan-ECH NIPs described above, the adsorption of the PFCs on the surface of amine grafted biomaterials occurred via hydrophobic interactions and ion exchange, since such PFCs exist in their anion form when pH > pK_a_ in aqueous solution. Selected physicochemical properties of PFOA, PFOS, and PFBA are compared in Table 1 [92,93]. The materials design strategy by Deng *et al*. was based on creating pore structures by grafting long polymers containing quaternary ammonium ions onto functionalized nonporous biomaterials. Hence, the biomaterial adsorbents may have enhanced pore structure and variable surface chemistry. The enhanced pore structure of the NIP biomaterial adsorbents was attributed to the presence of long polymer brushes on the surface of the fibrous cotton that were functionalized with quaternary ammonium cation moieties at high density to afford increased uptake of PFCs. The NIP biomaterial adsorbents exhibited good sorption up to 3.3 mmol.g^−1^ with equilibration times that range from 4 to 12 h. In contrast, longer time was required for the hydrophobic and larger PFOS molecule (*cf*. Table 1) as it diffuses through the long polymer brushes to the active adsorption sites on the surface. The occurrence of favorable adsorbent-adsorbate electrostatic interactions between anionic guests and the surface of aminated NIP adsorbents were reported by Zhang *et al*. [89] and Deng *et al*. [90,91]. The utility of such modified biopolymers may pose challenges to the stability and regeneration of such materials depending on the pH of the effluent water. Furthermore, the adsorbent materials showed complete saturation at relatively low equilibrium concentration values compared to other polymer adsorbents, and may be due to the limited pore structure properties from the lack of pre-organized binding sites. The use of MIPs may overcome the longer equilibration times since the active sites are more readily accessible due to the availability of pre-organized binding sites. The latter relates to the production of adsorbents with variable surface area and pore structure properties due to the use of molecular templates. In addition, molecular templates such as a porogen solvent and/or macromolecular building block afford materials with enhanced surface area and textural properties with nano- to micro-scale porosity.

**Table 1:** Physicochemical Properties of PFOA, PFOS, and PFBA. Reproduced with permission from [94], Copyright 2014, American Chemical Society.

Molecular Formula and Structure *	**PFOA**	**PFOS**	**PFBA**
	C_8_HF_15_O_2_	C_8_HF_17_O_3_S	C_4_HF_7_O_2_
	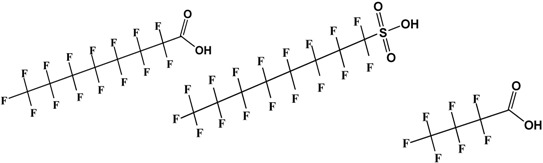		
Molecular weight (g/moL)	414	500	214
Solubility (g/L, 25 °C)	3.4 ^a^	0.57	High
Melting Point (°C)	45–55	45–54	−17.5
Boiling Point (°C)	188	188–192	120
pK_a_	2.5	0.14	0.08–0.4
cmc (mmol/L)	8.5–10	2.0	No data
Vapor pressure (mmHg, 25 °C)	0.017	2.48 × 10^−6^	10

* The conformation and stereochemistry of the PFCs are not accurately depicted and are meant to show the carbon skeletal framework. ^a^ 9.5 g/L (25 °C) has also been reported.

### 3.2. Conventional MIP Adsorbents

**Figure 2:**
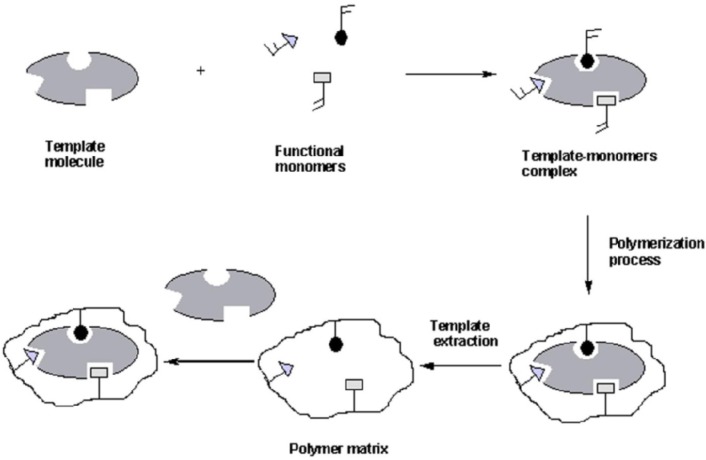
Preparation of molecularly imprinted polymers with functional monomers and a template molecule. Reproduced with permission from [95], Copyright 2011, MDPI AG.

Molecular imprinting technology is a well-established method for the preparation of polymers that possess specific molecular recognition properties [95,96,97,98,99,100,101,102,103]. Variable design strategies for conventional MIPs that employ molecular templates, one or more functional monomers, and a polymerization initiator to yield a cross-linked 3-D polymer network have been reviewed [104,105,106]. Thorough washing of the template molecules usually results in material frameworks that have microcavity imprints with a 3-D topology that is complementary in geometry and chemical functionality to the molecular template. Two strategies for preparing MIPs have been described in the literature that are relevant to the preparation of CD-MIPs (*cf.* Section 3.3) and a generalized scheme for the preparation of a conventional MIP is illustrated in Figure 2.

**Figure 3:**
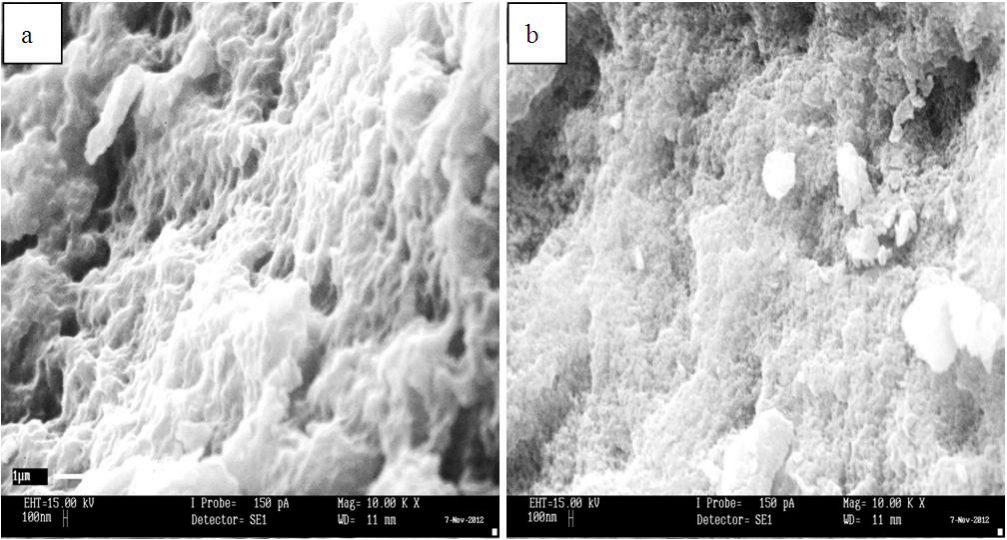
SEM micrographs of (**a**) methacrylic acid (MAA)-βCD molecularly imprinted polymer (MIP) and (**b**) MAA-βCD non-imprinted polymer (NIP) adsorbents. Reproduced with permission from [109], Copyright 2014, MDPI AG.

**Figure 4:**
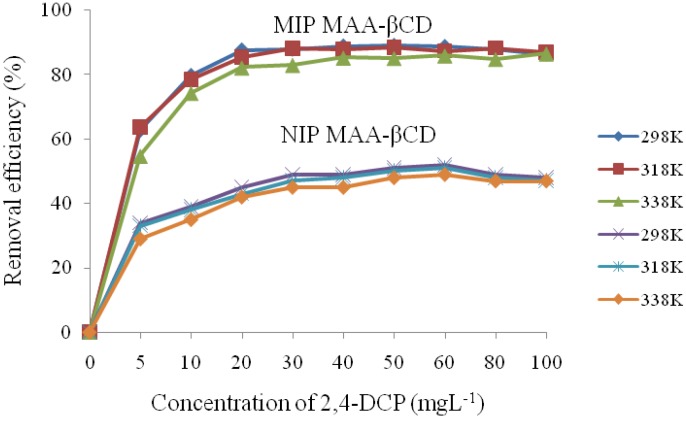
Adsorption of 2,4-Dichlorophenol onto MIP MAA-βCD and NIP MAA-βCD under different temperatures. Reproduced with permission from [109], Copyright 2014, MDPI AG.

Early reports on MIP-based adsorbents for the recognition of PFCs was described by Yu, Deng and coworkers [107,108]. The first MIP for recognition of PFOS was prepared by cross-linking chitosan with epichlorohydrin in the presence of PFOS as the molecular template [107]. The materials design strategy was based on a number of factors: (i) use of a chitosan biopolymer to prepare the MIP through a two-step cross-linking reaction, and (ii) increasing the amount of template and optimization of synthetic conditions (pH, temperature, solvent, *etc.*) to achieve an optimal imprinting yield. While factor (i) was aimed at enhancing the surface area and modifying the morphological properties, the second factor was used to ensure efficient molecular recognition by enhancing uptake through increasing the number of active sites. The chitosan-based MIPs reported by Yu *et al*. [107] generally showed greater sorption (~2.9 mmol.g^−1^) compared to NIP materials (~2.4 mmol.g^−1^). The NIPs were prepared similarly but in the absence of a template and could be re-used several times. The difference in surface morphology between the MIPs and NIPs was examined using SEM (*cf*. Figure 2 [107]). The structural variability of the materials was suggested to account for the variable uptake properties, where the MIPs were observed to be greater than the NIPs (*cf*. Figure 3 [107]). The SEM micrographs revealed well-defined pores in the nanometer range on the MIP surface that were formed during the imprinting step that were retained upon removal of the template. In contrast, the NIP surface showed roughness without defined pore structure (*cf*. Figure 2, [107]). Similar results were reported elsewhere [109] for a MIP that was formed using a methacrylic acid functionalized β-CD (MAA-βCD). The MIP MAA-βCD exhibited greater removal efficiency for 2,4-dichlorophenol compared to NIP MAA-βCD. Figure 3 and Figure 4 depict the field emission SEM micrographs and the adsorption isotherms of the MAA-βCD adsorbents. The surface of the MIP MAA-βCD exhibits a more porous structure than the NIP MAA-βCD due to the imprinting of the specific binding sites. The MIP materials reported by Yu *et al*. [107] were successfully regenerated by deprotonation of the amine groups on chitosan in alkaline solution; however, such materials exhibited longer equilibrium times (~32 h), commensurate with the greater abundance of micropore domains on the MIP surface.

**Figure 5:**
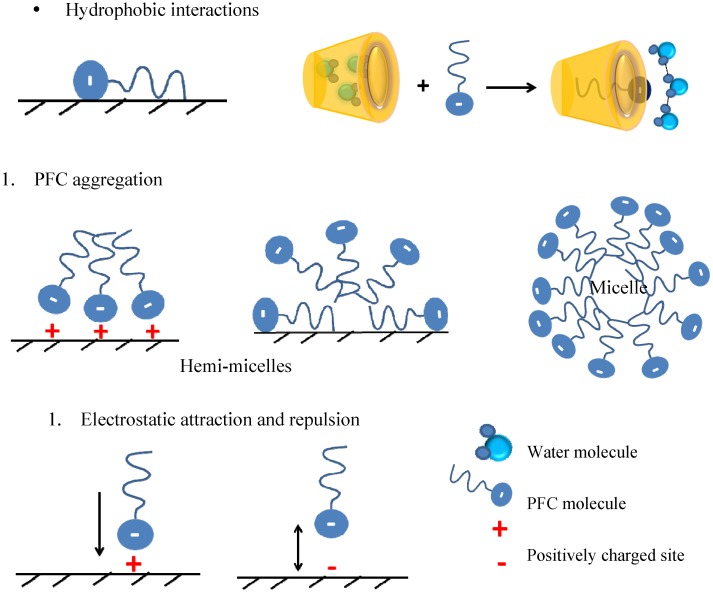
Schematic representation for (**a**) hydrophobic interactions; (**b**) PFC aggregation; and (c) electrostatic interactions (redrawn from [41]).

Deng *et al*. [108] prepared a series of MIPs by polymerization of 4-vinylpyridine in the presence of different PFC templates (PFOS and PFOA) and variable loading levels, cross-linkers, and solvents for recognition of PFOS. Similar to the study by Yu and coworkers [107], the effect of linker and solvent type, pH, ionic strength, and template loading on the imprinting efficiency was examined. As shown in Figure 4, the templation efficiency can be assumed to be affected by temperature. In the case of the chitosan-ECH materials reported by Yu (*cf*. Figure 3 in [107]), higher temperature (~40 °C) was shown to favor adsorption of PFC as a result of the imprint domain created by the molecular template. Other factors such as choice of cross-linker and solvent are of crucial importance to efficient imprinting. Similar to the chitosan-MIPs described above [107], the MIP materials prepared by Deng *et al.* [108] were superior compared to NIPs; however, the latter MIPs exhibited shorter equilibration times (<1 h). Pronounced binding was attributed to enhanced recognition between the template and the binding sites on the polymer surface. Deng *et al*. exploited the nitrogen atom functionalization of the vinylpyridine monomer which may undergo protonation at acidic conditions causing electrostatic interactions with the sulfonate group of PFOS. Subsequent formation of PFC micelles and hemi-micelles via the fluorocarbon tails at elevated adsorbate concentration, and may account for the overall increased adsorption capacity. The different types of interactions, *viz*. hydrophobic, electrostatic, and micelle formation are schematically shown in Figure 5. Note that electrostatic repulsion may be relevant at elevated solution pH (>5) since the protonated amine groups of the adsorbate may be deprotonated at pH conditions above the pK_a_. More recently, the use of macrocyclic porogens such as CDs in the preparation of MIPs has risen sharply [79,110,111,112,113,114,115] and is partly related to the unique properties of CD-MIPs. The general utility of such nano-structured polymer materials in pharmacology, catalysis, sensor technology and environmental science was reviewed by Crini [71].

### 3.3. CD-MIP Adsorbents

CD-MIPs possess some unique advantages for separation of chemical mixtures, and they have been studied for the molecular discrimination of pesticides, steroids, peptides, antibiotics, amino acids, and other organic compounds [99,100,101,102,103,110,111,112]. Several articles and reviews exist that describe the preparation of CD-MIPs for the separation of hydrocarbon mixtures [110,111,112,113]. However, reports on similar materials for targeting fluorocarbons are sparse due to challenges in the material design of conventional MIPs using molecular templates. Recently, Takayose *et al*. [116] circumnavigated this challenge by preparation of MIPs templated with PFOA from a fluorinated monomer and cross-linker for recognition of PFOA. The researchers took advantage of the fluorine-fluorine interactions between the template and the fluorinated cross-linker to create binding sites selective for fluorinated compounds. The retention factors of the fluorinated and non-fluorinated MIPs were compared using liquid chromatography with methanol-water mixture as an eluent. Although no uptake levels were reported, the fluorinated MIPs exhibited retention factors that were up to 10 times higher compared to similar values using non-fluorinated MIPs; which suggest that the retention of PFOA onto the polymer adsorbents is commensurate with the amount of fluorine functional groups on the cross-linker and/or monomer surface. The materials proposed by Takayose and co-workers are not expected to result in high and efficient uptake levels for PFCs because of the limited surface area and pore structure properties of these materials. Furthermore, such adsorbent materials may promote aggregation of PFC adsorbates via micelle formation as described in Figure 5b. Favorable interactions between the fluorinated surface and the headgroup of the fluorinated guest (Figure 5b) provide an alternate pathway to lower the Gibbs energy of hydration, as compared with processes driven by hydrophobic adsorption processes (*cf*. Figure 5a).

**Figure 6:**
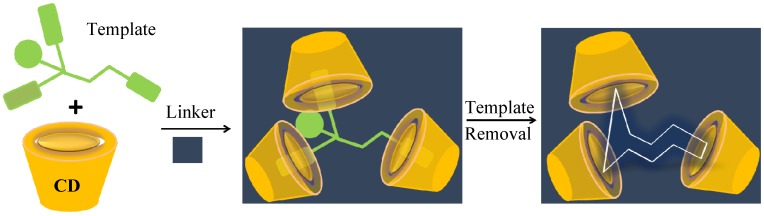
Schematic showing mechanism of CD-MIPs preparation using bulk imprinting technology (redrawn from [106]).

The incorporation of a macromolecular porogen such as β-CD in the material design of CD-MIPs represents a unique variation on the conventional preparation of MIPs using a conventional molecular template (*cf*. Figure 1 and Figure 2). The use of a pre-organized CD macrocycle serves as an intrinsic framework “porogen” due to the presence of the apolar cavity of the polysaccharide. Thus, the molecular recognition properties of such which contain CD have suitable binding sites (*i.e.*, porogen domains) since inclusion complexes can be formed with suitable-sized adsorbates. The presence of macromolecular porogens within a polymer framework presents an alternative design strategy where the physicochemical properties of the material can be controlled by an appropriate cross-linker, relative mole ratio of reagents, and reaction conditions [72]. For example, β-CD contains 21 hydroxyl groups that can be cross-linked to produce dimers, linear or branched polymers, and materials of different morphology (e.g., powders, gels, resins, and beads) and tunable physicochemical properties [110]. Moreover, the interior cavity of the CD host molecule provides a hydrophobic environment for molecular recognition of apolar guests according to *size-fit* considerations [80]. Piletsky *et al.* [99,100,101] and Komiyama *et al.* [102,103] described a balance of hydrophobic and electrostatic interactions are important for ligand recognition in CD-based MIPs, in addition to the importance of enthalpy-entropy compensation phenomena. Thus, template selective recognition can be envisaged to proceed via a combination of entropy-driven hydrophobic interactions of the template with the CD cavity, and enthalpy-driven interactions (e.g., electrostatic, ion pairing, H-bonding) with the cross-linker. Zhang *et al*. [104] and Folch-Cano *et al*. [105] have described two materials design strategies for the preparation of CD-MIPs; (1) bulk imprinting, and (2) surface imprinting. In Figure 6), the preparation of MIPs using bulk imprinting proceeds in solution by using a CD macrocycle with a cross-linker agent (e.g., diisocyanate) in the presence of a suitable template. Many CD-MIPs obtained by this method offer efficient molecular recognition toward small and large molecules that depend on the functionality of the CD monomer, nature of the cross-linking agent, and the reaction conditions (e.g., pH, temperature, solvent effects, *etc.*). In contrast to conventional MIPs, bulk imprinting of CD-based polymers for molecular recognition of small molecules may occur in the absence of a well-defined molecular template, provided that the size-fit topology and the hydrophile-lipophile balance of the CD host and the guest are maintained [79].

**Figure 7:**
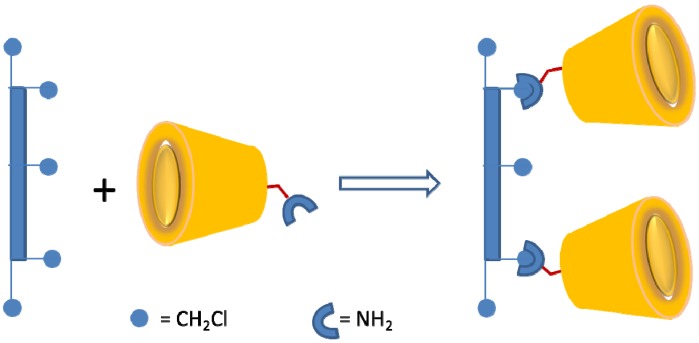
Schematic showing mechanism of CD-MIPs preparation using surface imprinting technology (redrawn from [115]).

In the case of surface imprinting (Figure 7), the technology employs an immobilized CD macromolecule onto a solid support such as silica-gel, alumina, or solid polymers. The molecular recognition of surface grafted CDs depends on the physicochemical properties of the solid supports since they dictate self-assembly of the CD molecules [104]. Figure 6 and Figure 7 illustrate the two strategies; bulk and surface imprinting methods. It should be noted that the relative orientation of the CD macrocycle occurs via the primary or secondary hydroxyl groups and affects the molecular recognition with various guests, in addition to steric effects that result from substitution of the CD annulus.

**Figure 8:**
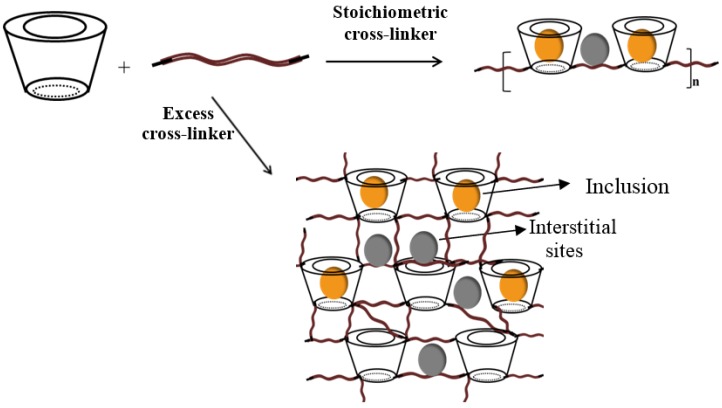
Association of a CD (cone) and a bi-functional reagent (e.g., diisocyanate cross-linker; wavy lines) to form linear (top) or branched (bottom) urethane based polymer materials, where guest (spheres) can bind in the inclusion sites or within the interstitial regions. Reproduced with permission from [118], Copyright 2014, University of Saskatchewan.

CD-MIPs that use surface imprinting technology for recognition of PFCs was reported elsewhere [114,115]. Bhattarai *et al*. [114] evaluated the molecular recognition of CD-MIPs formed by coating β-CD coating on silica for binding PFOA and other organic molecules. They prepared a series of CD-MIPs using different cross-linking agents (HDI and ECH), polymers and solvents. The CD-MIP with HDI as a cross-linking agent with DMSO as the solvent was observed to exhibit greater PFOA removal efficiency. The result was attributed to the amount of β-CD loading onto the silica support. Host-guest inclusion complex formation between CD and PFOA occurs via weak hydrophobic and van der Waals interactions as the primary mode of sorption for PFOA (*cf*. Figure 5a), and secondary H-bonding interactions with the hydroxyl groups of the CD. Moreover, the HDI linker agent may provide additional pore structure properties within the polymer framework and secondary H-bond acceptor or donor binding sites for PFOA [72,79,117]. The CD-MIPs prepared by Bhatarrai and coworkers were successfully regenerated using methanol. PFOA is relatively soluble in methanol and the solvent efficiently displaces the bound guest due to favorable solvent interactions with the host and guest, resulting in regeneration of the CD-MIP over successive cycles. Kawano *et al*. [115] reported the preparation of CD-MIPs with β-CD supported onto polystyrene particles for recognition of PFOA and PFOS (*cf*. Figure 7). Similar to the CD-MIPs reported by Bhattarai *et al.* [114], the molecular recognition of CD-polystyrene MIPs for PFOA was achieved via hydrophobic interactions within the CD cavity with an overall uptake in the range of 0.1 to 0.5 mmol.g^−1^. As for the fluorinated materials reported by Takayose and coworkers [116], the reduced uptake of the surface grafted CD-MIPs is related to limited surface area, pore structure and morphological properties of the 2-D surface. In a recent study by Karoyo and Wilson [79], polymers with unique structure were prepared by reaction of β-CD with a bi-functional HDI linker agent to prepare bulk CD-MIPs for targeting PFOA and PFOS in aqueous solution. The preparation of the bulk CD-HDI MIPs is illustrated by Figure 8.

**Figure 9:**
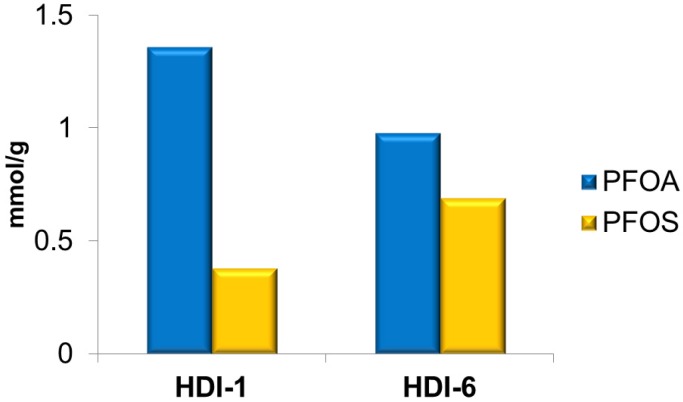
Adsorption of PFOA and PFOS on bulk CD-MIPs prepared using a cross-linker such as hexamethylene diisocyanate.

Cross-linked CD-MIPs offer unique structural features relative to surface grafted MIPs with respect to their pore structure, according to their micron versus nanometer dimensions. The physicochemical and pore structure properties of the cross-linked CD-MIPs are variable and depend on the reactions conditions, solvent and cross-linker agent, and the relative amounts of the cross-linker with respect to the CD macrocycle. For example, the relative ration of cross-linker ratios (one-, three-, and six-fold equivalents) with respect to mole content of CD give rise to cross-linked polymers denoted as HDI-1 (linear and water soluble), HDI-3 (branched and slightly soluble), and HDI-6 (branched and insoluble), respectively. The preparation of linear *vs*. globular CD-polymers for sorptive-based applications was reported by Karoyo and Wilson [79]. CD-polymers may undergo self-assembly and aggregation of the linear polymer (HDI-1), where it was reported to exhibit behavior observed that was characteristic of a “*smart material*” due to cooperative interactions [119]. The term, “*smart material*” refers to a polymer that exhibits changes in physical properties due to external stimuli such as concentration gradients of adsorbate speceis and/or external temperature variations. MIP-based “*smart materials*” are promising for future technology applications in chemical separations [118]. The adsorption of PFOA and PFOS onto the CD-based MIPs likely occurs via a combination of interactions as those mentioned in Figure 5; whereas, variable uptake levels in the range of ~0.9–1.4 mmol.g^−1^ were reported [79]. The molecular recognition and sorptive efficiency of these materials were tuned by adjusting the relative CD and linker composition, and the reaction conditions. As such, the materials design strategy enabled specific adsorbent-adsorbate interactions for tailored applications and facile adsorbent regeneration. Similar to the materials reported by Yu *et al.* [107] and Kawano *et al*. [115], the regeneration of the CD-HDI MIPs is possible with mild organic solvents such as methanol or aqueous alkaline media. The selectivity of cross-linked CD polymers was demonstrated even for structurally similar PFCs, such as PFOA and PFOS (*cf*. Figure 9). The results indicate that controlled cross-linking contributes significantly to molecular recognition of MIPs toward mixtures of compounds in water matrices. Supporting evidence of the fractionation of hydrocarbon carboxylic acid mixtures was achieved using a range of cross-linked CD polymers as demonstrated by Mohamed *et al*. [120].

## 4. Conclusions and Future Perspectives

Various types of adsorbent materials were evaluated for their ability to undergo efficient adsorption of perfluorochemicals (PFCs). In general, graphene-based carbonaceous adsorbent materials are often limited due to their inert character and the regeneration costs. Synthetic NIP materials may offer a better option compared to carbonaceous materials in terms of the synthetic diversity and improved uptake of PFCs. Challenges with the regeneration of the NIP materials may limit their use and field of application as molecular selective adsorbents. MIPs generally offer robust molecular recognition, particularly for PFCs, in accordance with the imprinting efficiency and the high binding affinity of PFCs with the macromolecular binding sites. Cross-linked biopolymers such as chitosan have significant sorptive uptake capacity compared to NIPs. However, longer equilibration times, binding sites with lesser preorganization, and polydisperse microporous materials that may limit optimal *size-fit* complementarity between the guest and polymer host binding sites. The key to materials design strategy for the efficient uptake of PFCs from aqueous solution is controlled by the textural properties (high surface area and porosity) along with appropriate surface functionality and preorganized binding sites. The rational use of porogens such as CDs and functional linker units in the materials design of MIPs addresses these considerations. The unique host-guest chemistry of CDs and their incorporation as macromolecular porogens into polymer frameworks offers several advantages:

1. *Size-fit* complementarity between the host binding sites and the guest at the nanometer scale and beyond;
2. Introduction of high affinity functional groups such as hydroxyl and ethers that possess high polarizability and greater affinity than graphene or carbonaceous materials that possess reduced levels of surface functional heteroatom groups; and
3. Greater ease of sorbent regeneration because relatively weak interactions are involved.

Future prospects for materials design strategy may involve the use of gel phase components over insoluble and less hydrated polymers as a way to enhance the uptake kinetics [121]. Gel phase materials are anticipated to increase the mass transfer kinetics due to the continuity of the solvent network at the gel-solvent interface, in contrast to poorly insoluble and less hydrated polymer materials. Composite materials that incorporate Lewis acid and/or base sites, high surface area and textural porosity, along with preorganized binding sites via macromolecular porogens represent a versatile future design strategy. Preorganized binding domains offer significant advantages over conventional imprinting technology used in MIPs since CD macrocycles are well-defined and maintain their structural integrity over diverse conditions. The limitations of molecular template-based MIPs was recently reviewed [122] and further insight was provided as to why solvent regeneration attenuates the uptake capacity. For example, successive washing of MIPs may result in the collapse of the template imprint sites for specific solvent systems. As well, there are challenges associated with an optimal monomer and cross-linker system in the polymerization process since solvation phenomena can attenuate the available imprinting sites for the template. The complexity of designing MIPs in aqueous solution was evidenced in a recent study [123] where structural modification of cross-linked cyclodextrin polymers was achieved under thermodynamic versus kinetically controlled conditions.

## References

[1] Petitpierre-Sauvain A. (2008). The Basel, Rotterdam and Stockholm Conventions on Chemicals and Wastes—Regulation, Sound Management and Governance. Trade, the Environment and Technology Transfer.

[2] Fujii S., Polprasert C., Tanaka S., Lien N.P.H., Qiu Y. (2007). New POPs in the water environment: Distribution, bioaccumulation and treatment of perfluorinated compounds—A review paper. J. Water Supply Res. Technol.-AQUA.

[3] Giesy J.P., Kannan K. (2002). Perfluorochemical surfactants in the environment. Environ. Sci. Technol..

[4] Post G.B., Cohn P.D., Cooper K.R. (2012). Perfluorooctanoic acid (PFOA), an emerging drinking water contaminant: A critical review of recent literature. Environ. Res..

[5] Liou J.S.C., Szostek B., DeRito C.M., Madsen E.L. (2010). Investigating the biodegradability of perfluorooctanoic acid. Chemosphere.

[6] Becker A.M., Suchan M., Gerstmann S., Frank H. (2010). Perfluorooctanoic acid and perfluorooctane sulfonate released from a waste water treatment plant in Bavaria, Germany. Environ. Sci. Pollut. Res..

[7] Zareitalabad P., Siemens J., Hamer M., Amelung W. (2013). Perfluorooctanoic acid (PFOA) and perfluorooctanesulfonic acid (PFOS) in surface waters, sediments, soils and wastewater—A review on concentrations and distribution coefficients. Chemosphere.

[8] Fernández-Sanjuan M., Meyer J., Damásio J., Faria M., Barata C., Lacorte S. (2010). Screening of Perfluorinated Chemicals (PFCs) in Various Aquatic Organisms. Anal. Bioanal. Chem..

[9] Zainuddin K., Zakaria M.P., Al-Odaini N.A., Bakhtiari A.R., Latif P.A. (2012). Perfluorooctanoic Acid (PFOA) and Perfluorooctane Sulfonate (PFOS) in Surface Water From the Langat River, Peninsular Malaysia. Environ. Forensics.

[10] Fei C., McLaughlin J.K., Lipworth L., Olsen J. (2009). Maternal levels of perfluorinated chemicals and subfecundity. Hum. Reprod..

[11] Lau C., Butenhoff J.L., Rogers J.M. (2004). The developmental toxicity of perfluoroalkyl acids and their derivatives. Toxicol. Appl. Pharmacol..

[12] Lau C., Thibodeaux J.R., Hanson R.G., Narotsky M.G., Rogers J.M., Lindstrom A.B., Strynar M.J. (2006). Effects of perfluorooctanoic acid exposure during pregnancy in the mouse. Toxicol. Sci..

[13] Butenhoff J.L., Kennedy G.L., Frame S.R., O’Connor J.C., York R.G. (2004). The reproductive toxicology of ammonium perfluorooctanoate (APFO) in the rat. Toxicology.

[14] Case M.T., York R.G., Christian M.S. (2001). Rats and rabit oral developmental taxicological studies with two perfluorinated compounds. Int. J. Toxicol..

[15] Kawashima Y., Kobayashi H., Miura H., Kozuka H. (1995). Characterization of hepatic responses of rat to administration of perfluorooctanoic and perfluorodecanoic acids at low levels. Toxicology.

[16] Kudo N., Iwase Y., Okayachi H., Yamakawa Y., Kawashima Y. (2005). Induction of hepatic peroxisome proliferation by 8-2 telomer alcohol feeding in mice: Formation of perfluorooctanoic acid in the liver. Toxicol. Sci..

[17] Yang Q., Abedi-Valugerdi M., Xie Y., Zhao X.Y., Möller G., Dean Nelson B., DePierre J.W. (2002). Potent suppression of the adaptive immune response in mice upon dietary exposure to the potent peroxisome proliferator, perfluorooctanoic acid. Int. Immunopharmacol..

[18] Peden-Adams M.M., Keller J.M., Eudaly J.G., Berger J., Gilkeson G.S., Keil D.E. (2008). Suppression of humoral immunity in mice following exposure to perfluorooctane sulfonate. Toxicol. Sci..

[19] Dewitt J.C., Copeland C.B., Luebke R.W. (2009). Suppression of humoral immunity by perfluorooctanoic acid is independent of elevated serum corticosterone concentration in mice. Toxicol. Sci..

[20] Guruge K.S., Yeung L.W.Y., Yamanaka N., Miyazaki S., Lam P.K.S., Giesy J.P., Jones P.D., Yamashita N. (2006). Gene expression profiles in rat liver treated with perfluorooctanoic acid (PFOA). Toxicol. Sci..

[21] Kennedy G.L., Butenhoff J.L., Olsen G.W., O’Connor J.C., Seacat A.M., Perkins R.G., Biegel L.B., Murphy S.R., Farrar D.G. (2004). The toxicology of perfluorooctanoate. Crit. Rev. Toxicol..

[22] Gilliland F.D., Mandel J.S. (1993). Mortality among employees of a perfluorooctanoic acid production plant. J. Occup. Environ. Med..

[23] Vierke L., Staude C., Biegel-Engler A., Drost W., Schulte C. (2012). Perfluorooctanoic acid (PFOA)-main concerns and regulatory developments in Europe from an environmental point of view. Environ. Sci. Eur..

[24] Yang K.-H., Lin Y.-C., Fang M.-D., Wu C.-H., Panchangam S.C., Hong P.-K.A., Lin C.-F. (2013). Sorption of perfluorooctanoic acid (PFOA) onto sediment in the presence of dissolved natural organics. Sep. Sci. Technol..

[25] Giesy J.P., Naile J.E., Khim J.S., Jones P.D., Newsted J.L. (2010). Aquatic toxicology of perfluorinated chemicals. Rev. Environ. Contam. Toxicol..

[26] Kissa E. (2001). Fluorinated Surfacts and Repellents.

[27] Ding G., Peijnenburg W.J.G.M. (2013). Physicochemical properties and aquatic toxicity of poly-and perfluorinated compounds. Crit. Rev. Environ. Sci. Technol..

[28] Stahl T., Mattern D., Brunn H. (2011). Toxicology of perfluorinated compounds. Environ. Sci. Eur..

[29] Shi Y., Pan Y., Wang J., Cai Y. (2012). Distribution of perfluorinated compounds in water, sediment, biota and floating plants in Baiyangdian Lake, China. J. Environ. Monit..

[30] Kwadijk C.J.A.F., Korytár P., Koelmans A.A. (2010). Distribution of perfluorinated compounds in aquatic systems in the netherlands. Environ. Sci. Technol..

[31] Naile J.E., Khim J.S., Hong S., Park J., Kwon B.O., Ryu J.S., Hwang J.H., Jones P.D., Giesy J.P. (2013). Distributions and bioconcentration characteristics of perfluorinated compounds in environmental samples collected from the west coast of Korea. Chemosphere.

[32] Guo W., Fung B.M., Christian S.D. (1992). NMR study of cyclodextrin inclusion of fluorocarbon surfactants in solution. Langmuir.

[33] Xing H., Lin S.-S., Yan P., Xiao J.-X., Chen Y.-M. (2007). NMR studies on selectivity of beta-cyclodextrin to fluorinated/hydrogenated surfactant mixtures. J. Phys. Chem. B.

[34] Wilson L.D., Verrall R.E. (1998). A ^1^H NMR study of cyclodextrin-hydrocarbon surfactant inclusion complexes in aqueous solutions. Can. J. Chem..

[35] Karoyo A.H., Borisov A.S., Wilson L.D., Hazendonk P. (2011). Formation of host-guest complexes of β-cyclodextrin and perfluorooctanoic acid. J. Phys. Chem. B.

[36] Zhang H., Hogen-Esch T.E., Boschet F., Margaillan A., Boschet F., Margaillan A. (1998). Complex formation of cyclodextrin- and perfluorocarbon- modified water-soluble polymers. Langmuir.

[37] Palepu R., Richardson J.E., Reinsborough V.C. (1989). Binding constants of .beta.-cyclodextrin/surfactant Inclusion by conductivity measurements. Langmuir.

[38] Palepu R., Reinsborough V.C. (1989). Solution inclusion complexes of cyclodextrins with sodium perfluorooctanoate. Can. J. Chem..

[39] Saint Aman Eric S.D. (1990). A conductimetric study of the association between cyclodextrins and surfactants-application to the electrochemical study of a mixed aqueous system: Substrate, cyclodextrin, surfactant. J. Colloid Interface Sci..

[40] Junquera E., Tardajos G., Aicart E. (1993). Effect of the presence of β-cyclodextrin on the micellization process of sodium dodecyl or sodium perfluoroctanoate in water. Langmuir.

[41] Du Z., Deng S., Bei Y., Huang Q., Wang B., Huang J., Yu G. (2014). Adsorption behavior and mechanism of perfluorinated compounds on various adsorbents—A review. J. Hazard. Mater..

[42] Richardson S., Ternes T. (2014). Water analysis: Emerging contaminants and current issues. Anal. Chem..

[43] Moriwaki H., Takagi Y., Tanaka M., Tsuruho K., Okitsu K., Maeda Y. (2005). Sonochemical decomposition of perfluorooctane sulfonate and perfluorooctanoic acid. Environ. Sci. Technol..

[44] Yamamoto T., Noma Y., Sakai S.I., Shibata Y. (2007). Photodegradation of perfluorooctane sulfonate by UV irradiation in water and alkaline 2-propanol. Environ. Sci. Technol..

[45] Zhao H., Gao J., Zhao G., Fan J., Wang Y., Wang Y. (2013). Fabrication of novel SnO_2_-Sb/carbon aerogel electrode for ultrasonic electrochemical oxidation of perfluorooctanoate with high catalytic efficiency. Appl. Catal. B Environ..

[46] Ochoa-Herrera V., Sierra-Alvarez R. (2008). Removal of perfluorinated surfactants by sorption onto granular activated carbon, zeolite and sludge. Chemosphere.

[47] Yu Q., Zhang R., Deng S., Huang J., Yu G. (2009). Sorption of perfluorooctane sulfonate and perfluorooctanoate on activated carbons and resin: Kinetic and isotherm study. Water Res..

[48] Qu Y., Zhang C., Li F., Bo X., Liu G., Zhou Q. (2009). Equilibrium and kinetics study on the adsorption of perfluorooctanoic acid from aqueous solution onto powdered activated carbon. J. Hazard. Mater..

[49] Hansen M.C., Børresen M.H., Schlabach M., Cornelissen G. (2010). Sorption of perfluorinated compounds from contaminated water to activated carbon. J. Soils Sediments.

[50] Li X., Chen S., Quan X., Zhang Y. (2011). Enhanced adsorption of PFOA and PFOS on multiwalled carbon nanotubes under electrochemical assistance. Environ. Sci. Technol..

[51] Carter K.E., Farrell J. (2010). Removal of perfluorooctane and perfluorobutane sulfonate from water via carbon adsorption and ion exchange. Sep. Sci. Technol..

[52] Senevirathna S.T.M.L.D., Tanaka S., Fujii S., Kunacheva C., Harada H., Shivakoti B.R., Okamoto R. (2010). A comparative study of adsorption of perfluorooctane sulfonate (PFOS) onto granular activated carbon, ion-exchange polymers and non-ion-exchange polymers. Chemosphere.

[53] Zhao D., Cheng J., Vecitis C.D., Hoffmann M.R. (2011). Sorption of perfluorochemicals to granular activated carbon in the presence of ultrasound. J. Phys. Chem. A.

[54] Deng S., Zhang Q., Nie Y., Wei H., Wang B., Huang J., Yu G., Xing B. (2012). Sorption mechanisms of perfluorinated compounds on carbon nanotubes. Environ. Pollut..

[55] Chularueangaksorn P., Tanaka S., Fujii S., Kunacheva C. (2014). Batch and column adsorption of perfluorooctane sulfonate on anion exchange resins and granular activated carbon. J. Appl. Polym. Sci..

[56] Yao Y., Volchek K., Brown C.E., Robinson A., Obal T. (2014). Comparative study on adsorption of perfluorooctane sulfonate (PFOS) and perfluorooctanoate (PFOA) by different adsorbents in water. Water Sci. Technol..

[57] Jones D.A., Lelyveld T.P., Mavrofidis S.D., Kingman S.W., Miles N.J. (2002). Microwave heating applications in environmental engineering—a review. Resour. Conserv. Recycl..

[58] Salvador F., Jiménez C.S. (1996). A new method for regenerating activated carbon by thermal desorption with liquid water under subcritical conditions. Carbon N. Y..

[59] Chen M.S., Wang J.H. (2006). Microwave regeneration of activated carbon containing toluene. Environ. Pollut. Control Technol. Equip..

[60] Zhang H., Ye L., Zhong H. (2002). Regeneration of phenol-saturated activated carbon in an electrochemical reactor. J. Chem. Technol. Biotechnol..

[61] Mundale V.D., Joglekar H.S., Kalam A., Joshi J.B. (1991). Regeneration of spent activated carbon by wet air oxidation. Can. J. Chem. Eng..

[62] Yu J.G., Zhao X.H., Yang H., Chen X.H., Yang Q., Yu L.Y., Jiang J.H., Chen X.Q. (2014). Aqueous adsorption and removal of organic contaminants by carbon nanotubes. Sci. Total Environ..

[63] Crini G. (2003). Studies on adsorption of dyes on beta-cyclodextrin polymer. Bioresour. Technol..

[64] Crini G., Peindy H.N., Gimbert F., Robert C. (2007). Removal of C.I. Basic Green 4 (Malachite Green) from aqueous solutions by adsorption using cyclodextrin-based adsorbent: Kinetic and equilibrium studies. Sep. Purif. Technol..

[65] Crini G., Bertini S., Torri G., Naggi A., Sforzini D., Vecchi C., Janus L., Lekchiri Y., Morcellet M. (1998). Sorption of aromatic compounds in water using insoluble cyclodextrin polymers. J. Appl. Polm. Sci..

[66] Mamba B.B., Krause R.W., Malefetse T.J., Nxumalo E.N. (2007). Monofunctionalized cyclodextrin polymers for the removal of organic pollutants from water. Environ. Chem. Lett..

[67] Morin-Crini N., Crini G. (2012). Environmental applications of water-insoluble β-cyclodextrin–epichlorohydrin polymers. Prog. Polym. Sci..

[68] Murai S., Imajo S., Inumaru H., Takahashi K., Hattori K. (1997). Adsorption and recovery of ionic surfactants by beta-cyclodextrin polymer. J. Colloid Interface Sci..

[69] Asanuma H., Kakazu M., Shibata M., Hishiya T., Komiyama M. (1998). Synthesis of molecularly imprinted polymers of beta-cyclodextrin for the efficient recognition of cholesterol. Supramol. Sci..

[70] Van de Manakker F., Vermonden T., van Nostrum C.F., Hennink W.E. (2009). Cyclodextrin-based polymeric materials: Synthesis, properties, and pharmaceutical/biomedical applications. Biomacromolecules.

[71] Crini G., Morcellet M. (2002). Synthesis and applications of adsorbents containing cyclodextrins. J. Sep. Sci..

[72] Mohamed M.H., Wilson L.D., Headley J.V. (2011). Design and characterization of novel  β-cyclodextrin based copolymer materials. Carbohydr. Res..

[73] Mohamed M.H., Wilson L.D., Headley J.V., Peru K.M. (2011). Sequestration of naphthenic acids from aqueous solution using β-cyclodextrin-based polyurethanes. Phys. Chem. Chem. Phys..

[74] Pratt D.Y., Wilson L.D., Kozinski J.A., Mohart A.M. (2010). Preparation and sorption studies of β-cyclodextrin/epichlorohydrin copolymers. J. Appl. Polym. Sci..

[75] Wilson L.D., Guo R. (2012). Preparation and sorption studies of polyester microsphere copolymers containing β-cyclodextrin. J. Colloid Interface Sci..

[76] Wilson L.D., Mohamed M.H., Headly J.V. (2014). Novel materials fo environmental remediation of oil sands contaminants. Rev. Env. Heal..

[77] Kryscio D.R., Peppas N.A. (2012). Critical review and perspective of macromolecularly imprinted polymers. Acta Biomater..

[78] Szejtli J., Osa T. (1996). Comprehensive Supramolecular Chemistry.

[79] Karoyo A.H., Wilson L.D. (2013). Tunable macromolecular-based materials for the adsorption of perfluorooctanoic and octanoic acid anions. J. Colloid Interface Sci..

[80] Palepu R., Reinsborough V.C. (1988). Surfactant-yclodextrin interactions by conductance measurements. Can. J. Chem..

[81] Del Valle E.M.M. (2004). Cyclodextrins and their uses: A review. Process Biochem..

[82] Szejtli J. (1997). Utilization of cyclodextrins in industrial products and processes. J. Mater. Chem..

[83] Steed J.W., Atwood J.L. (2009). Supramolecular Chemistry.

[84] Parker K.M., Stalcup A.M. (2008). Affinity capillary electrophoresis and isothermal titration calorimetry for the determination of fatty acid binding with beta-cyclodextrin. J. Chromatogr. A.

[85] Wenz G. (1994). Cyclodextrins as building-blocks for supramolecular structures and functional units. Angew. Chem. Int. Ed. Engl..

[86] Qaqish S.E., Urquhart S.G., Lanke U., Brunet S.M.K., Paige M.F. (2009). Phase separation of palmitic acid and perfluorooctadecanoic acid in mixed langmuir-blodgett monolayer films. Langmuir.

[87] Asakawa T., Amada K., Miyagishi S. (1997). Micellar immiscibility of lithium lithium tetradecyl sulfate mixture.

[88] Pesek J.J., Matyska M.T. (2000). SPE Sorbents and Formats. Solid-Phase Extraction: Principles, Techniques, and Applications.

[89] Zhang Q., Deng S., Yu G., Huang J. (2011). Removal of perfluorooctane sulfonate from aqueous solution by crosslinked chitosan beads: Sorption kinetics and uptake mechanism. Bioresour. Technol..

[90] Deng S., Zheng Y.Q., Xu F.J., Wang B., Huang J., Yu G. (2012). Highly efficient sorption of perfluorooctane sulfonate and perfluorooctanoate on a quaternized cotton prepared by atom transfer radical polymerization. Chem. Eng. J..

[91] Deng S., Niu L., Bei Y., Wang B., Huang J., Yu G. (2013). Adsorption of perfluorinated compounds on aminated rice husk prepared by atom transfer radical polymerization. Chemosphere.

[92] Burns D.C., Ellis D.A., Li H., Mcmurdo C.J., Webster E. (2008). Experimental pK_a_ determination for perfluorooctanoic acid (PFOA) and the potential impact of pK_a_ concentration dependence on laboratory-measured partitioning phenomena and environmental modeling. Environ. Sci. Technol..

[93] Hoffmann H., Würtz J. (1997). Unusual phenomena in perfluorosurfactants. J. Mol. Liq..

[94] Karoyo A.H., Sidhu P., Wilson L.D., Hazendonk P. (2014). Characterization and dynamic properties for the solid inclusion complexes of β-cyclodextrin and perfluorobutyric acid. J. Phys. Chem. C.

[95] Vasapollo G., Del Sole R., Mergola L., Lazzoi M.R., Scardino A., Scorrano S., Mele G. (2011). Molecularly imprinted polymers: Present and future prospective. Int. J. Mol. Sci..

[96] Cheong W.J., Yang S.H., Ali F. (2013). Molecular imprinted polymers for separation science: A review of reviews. J. Sep. Sci..

[97] Qiao F., Sun H., Yan H., Row K.H. (2006). Molecularly imprinted polymers for solid phase extraction. Chromatographia.

[98] Lasáková M., Jandera P. (2009). Molecularly imprinted polymers and their application in solid phase extraction. J. Sep. Sci..

[99] Piletsky S.A., Andersson H.S., Nicholls I.A. (1998). The rational use of hydrophobic effect-based recognition in molecularly imprinted polymers. J. Mol. Recognit..

[100] Piletsky S.A., Andersson H.S., Nicholls I.A. (1999). Combined hydrophobic and electrostatic interaction-based recognition in molecularly imprinted polymers. Macromolecules.

[101] Piletsky S.A., Andersson H.S., Nicholls I.A. (2005). On the role of electrostatic interactions in the enantioselective recognition of phenylalanine in molecularly imprinted polymers incorporating β-cyclodextrin. Polym. J..

[102] Song S.H., Shirasaka K., Katayama M., Nagaoka S., Yoshihara S., Osawa T., Sumaoka J., Asanuma H., Komiyama M. (2007). Recognition of solution structures of peptides by molecularly imprinted cyclodextrin polymers. Macromolecules.

[103] Asanuma H., Hishiya T., Komiyama M. (2004). Efficient separation of hydrophobic molecules by molecularly imprinted cyclodextrin polymers. J. Incl. Phenom..

[104] Zhang J., Shen X., Chen Q. (2011). Separation processes in the presence of cyclodextrins using molecular imprinting technology and ionic liquid cooperating approach. Curr. Org. Chem..

[105] Folch-Cano C., Yazdani-Pedram M., Olea-Azar C. (2014). Inclusion and functionalization of polymers with cyclodextrins: Current applications and future prospects. Molecules.

[106] Kyzas G.Z., Lazaridis N.K., Bikiaris D.N. (2013). Optimization of chitosan and β-cyclodextrin molecularly imprinted polymer synthesis for dye adsorption. Carbohydr. Polym..

[107] Yu Q., Deng S., Yu G. (2008). Selective removal of perfluorooctane sulfonate from aqueous solution using chitosan-based molecularly imprinted polymer adsorbents. Water Res..

[108] Deng S., Shuai D., Yu Q., Huang J., Yu G. (2009). Selective sorption of perfluorooctane sulfonate on molecularly imprinted polymer adsorbents. Front. Environ. Sci. Eng. China.

[109] Surikumaran H., Mohamad S., Sarih N.M. (2014). Molecular imprinted polymer of methacrylic acid functionalised β-cyclodextrin for selective removal of 2,4-dichlorophenol. Int. J. Mol. Sci..

[110] Asanuma H., Kakazu M., Shibata M., Hishiya T. (1997). Molecularly imprinted polymer of β-cyclodextrin for the efficient recognition of cholesterol. Chem. Commun..

[111] Xu Z.F., Wen G., Kuang D.Z., Zhang F.X., Tang S.P. (2013). Selective separation of deltamethrin by molecularly imprinted polymers using a β-cyclodextrin derivative as the functional monomer. J. Environ. Sci. Heal. B.

[112] Asanuma H., Akiyama T., Kajiya K., Hishiya T., Komiyama M. (2001). Molecular imprinting of cyclodextrin in water for the recognition of nanometer-scaled guests. Anal. Chim. Acta.

[113] Qin L., He X.W., Li W.Y., Zhang Y.K. (2008). Molecularly imprinted polymer prepared with bonded β-cyclodextrin and acrylamide on functionalized silica gel for selective recognition of tryptophan in aqueous media. J. Chromatogr. A.

[114] Bhattarai B., Muruganandham M., Suri R.P.S. (2014). Development of high efficiency silica coated β-cyclodextrin polymeric adsorbent for the removal of emerging contaminants of concern from water. J. Hazard. Mater..

[115] Kawano S., Kida T., Takemine S., Matsumura C., Nakano T., Kuramitsu M., Adachi K., Akashi M. (2013). Efficient removal and recovery of perfluorinated compounds from water by surface-tethered β-cyclodextrins on polystyrene particles. Chem. Lett..

[116] Takayose M., Nishimoto K., Matsui J. (2012). A fluorous synthetic receptor that recognizes perfluorooctanoic acid (PFOA) via fluorous interaction obtained by molecular imprinting. Analyst.

[117] Ma M., Li D. (1999). New organic nanoporous polymers and their inclusion complexes. Chem. Mater..

[118] Karoyo A.H. (2014). Structural Studies of Supramolecular Host-Guest Systems. Ph.D. Thesis.

[119] Deng W., Yamaguchi H., Takashima Y., Harada A. (2007). A chemical-responsive supramolecular hydrogel from modified cyclodextrins. Angew. Chem. Int. Ed..

[120] Mohamed M.H., Wilson L.D., Headley J.V. (2013). Tunable polymeric sorbent materials for fractionation of model naphthenates. J. Phys. Chem. B.

[121] Chen G., Jiang M. (2011). Cyclodextrin-based inclusion complexation bridging supramolecular chemistry and macromolecular self-assembly. Chem. Soc. Rev..

[122] Mohamed M.H., Wilson L.D. (2012). Porous copolymer resins: Tuning pore structure and surface area with non reactive porogens. Nanomaterials.

[123] Mohamed M.H., Wilson L.D., Headley J.V. (2015). Tuning the physicochemical properties of β-cyclodextrin based polyurethanes via cross-linking conditions. Microporous Mesoporous Mater..

